# Genome-Wide Characterization of Insertion and Deletion Variation in Chicken Using Next Generation Sequencing

**DOI:** 10.1371/journal.pone.0104652

**Published:** 2014-08-18

**Authors:** Yiyuan Yan, Guoqiang Yi, Congjiao Sun, Lujiang Qu, Ning Yang

**Affiliations:** Department of Animal Genetics and Breeding, College of Animal Science and Technology, China Agricultural University, Beijing, China; The University of Hong Kong, Hong Kong

## Abstract

Insertion and deletion (INDEL) is one of the main events contributing to genetic and phenotypic diversity, which receives less attention than SNP and large structural variation. To gain a better knowledge of INDEL variation in chicken genome, we applied next generation sequencing on 12 diverse chicken breeds at an average effective depth of 8.6. Over 1.3 million non-redundant short INDELs (1–49 bp) were obtained, the vast majority (92.48%) of which were novel. Follow-up validation assays confirmed that most (88.00%) of the randomly selected INDELs represent true variations. The majority (95.76%) of INDELs were less than 10 bp. Both the detected number and affected bases were larger for deletions than insertions. In total, INDELs covered 3.8 Mbp, corresponding to 0.36% of the chicken genome. The average genomic INDEL density was estimated as 0.49 per kb. INDELs were ubiquitous and distributed in a non-uniform fashion across chromosomes, with lower INDEL density in micro-chromosomes than in others, and some functional regions like exons and UTRs were prone to less INDELs than introns and intergenic regions. Nearly 620,253 INDELs fell in genic regions, 1,765 (0.28%) of which located in exons, spanning 1,358 (7.56%) unique Ensembl genes. Many of them are associated with economically important traits and some are the homologues of human disease-related genes. We demonstrate that sequencing multiple individuals at a medium depth offers a promising way for reliable identification of INDELs. The coding INDELs are valuable candidates for further elucidation of the association between genotypes and phenotypes. The chicken INDELs revealed by our study can be useful for future studies, including development of INDEL markers, construction of high density linkage map, INDEL arrays design, and hopefully, molecular breeding programs in chicken.

## Introduction

Chicken as one of the most important domestic animals not only provides essential proteins for human food industry, but also serves as an excellent biological model for many scientific researches [1]. Identifying genetic determinants of economically important traits or diseases is one of the main focuses of chicken genetic studies, which requires a comprehensive knowledge of DNA sequence variations as well as the development of numerous informative genetic markers. The near-complete chicken genome has made it possible to systematically study genetic variations. Up to now, several types of genetic variations have been identified across genomes, i.e. single nucleotide polymorphism (SNP), insertion and deletion (INDEL) and structural variation (SV). Studies in human show that INDEL is one of the main forms of genomic variations, with its occurrence in genome only second to SNP and even comparable to SNP in terms of affected bases [2], [3]. INDELs contribute substantially to genetic divergence both within and between species [4]–[7]. Besides, INDELs generally have a greater impact on gene functions than SNPs. Nearly 24% of the heritable disease mutations in human gene mutation database (HGMD) (http://www.hgmd.cf.ac.uk/ac/index.php) are INDELs. Many common human diseases are frequently caused by INDELs, such as cystic fibrosis [8] and Huntington's diseases [9]. In domestic animals, INDELs are also found to be responsible for a number of traits and diseases, such as double muscle trait [10] and factor XI deficiency [11] in cattle, immotile short-tail sperm defect in pig [12], and muscle mass in dog [13]. In chicken, INDELs of 9–15 bp in *PMEL17* gene are causative mutations for plumage color (Dominant white, Dun and Smoky) [14] and an INDEL mutation in the growth hormone receptor (GHR) gene causes sex-linked dwarfism [15]. Therefore, INDEL is gaining an increasing attention recently and has been extensively discovered and studied in a variety of species [3], [6], [16]–[20].

With the rapid advance of sequencing technology, considerable progresses have been made in INDEL discovery in chicken genome. Wong et al. [21] partially sequenced three chicken breeds by capillary sequencing and identified 2.8 million SNPs by aligning the resultant reads to the reference genome, and about 10% of these variations are actually INDELs. Based on these results, Brandstrom and Ellegren [5] estimated that INDELs in unique sequence were as 5% abundance as SNPs in chicken genome. In their efforts to detect selective sweeps, Rubin et al. [22] contributed almost 1,300 novel large deletions. Currently, more than 9 million variants have been deposited into the chicken SNP database, 438,865 of which are INDELs, accounting for 4.7% of all variants (ftp://ftp.ncbi.nih.gov/snp/organisms/chicken_9031/VCF/, updated in June 11, 2013). However, previous studies with human and other model organisms showed that INDELs accounted for 9–14% of all genetic polymorphisms [3], [23], . Therefore, the relatively small proportion of INDEL in chicken SNP database indicates that a large number of INDELs in chicken genome may not have been discovered.

Recently, Fan et al. [25] sequenced two chickens and identified over 600,000 INDELs per individual, which was a great contribution to the current variation database. However, for detailed examination and validation of INDEL variation in chicken genome, a larger and more representative collection of INDEL is still desired. To this end, we performed next generation sequencing (NGS) to detect genome-wide INDELs in 12 diverse chickens, representatives of both commercial and Chinese indigenous breeds. We focused on the identification of short INDELs (1–50 bp), which are the predominant forms of INDEL in the genome [5], [17], [26], [27]. We also examined the distribution of INDELs in chicken genome and their potential influence on gene functions, which would be helpful in deepening our understanding of chicken genome variation, developing INDEL markers, and elucidating the association between genetic variations and phenotypes in the future.

## Materials and Methods

### Ethics statements

The whole blood samples were collected from brachial veins of chickens by standard venipuncture. The whole procedure was performed according to the protocol approved by the Animal Care and Use Committee of China Agricultural University.

### Sample selection

Twelve female birds from 12 different chicken breeds were used in this study. Seven breeds, Beijing You (BY), Dongxiang (DX), Luxi game (LX), Shouguang (SG), Silkie (SK), Tibetan (TB) and Wenchang (WC), were Chinese indigenous. Four were commercial breeds, i.e., Cornish (CS), Rhode Island Red (RIR), White Leghorn (WL) and White Plymouth Rock (WR). A Red Jungle Fowl (RJF), the wild ancestor of domestic chickens, was also used. Birds from these breeds exhibit significant differences in appearance (comb type, skin and plumage color, etc.) and production performance (growth, egg production, feed consumption, etc.), and are believed to harbor extensive genetic diversity.

### Library construction and sequencing

Genomic DNAs were extracted from blood samples using standard phenol/chloroform extraction method. DNA concentration and purity were accessed on NanoDrop (Thermo Fisher Scientific Inc. Waltham, MA, USA), and the qualified DNAs were used for library construction. Two paired-end libraries were constructed for each individual, with an intended 10-fold depth (5-fold for each library). Genomic DNAs were sheared to yield an average size of 500 bp and then ligated to Illumina paired-end adaptors. After PCR amplification and purification, the resulting libraries were sequenced on an Illumina Hiseq 2000 sequencer (Illumina Inc., San Diego, CA, USA). Raw reads of 2×100 bp were generated for downstream analysis.

### Read mapping and variant calling

Chicken genome assembly (galGal4) was downloaded from UCSC Genome Browser website (http://hgdownload.soe.ucsc.edu/goldenPath/galGal4/bigZips/) [28]. In order to minimize mapping errors, we remove low quality reads with the help of NGS QC Toolkit [29] with default parameters. Considering the increasing error rate towards the end of reads due to the decay of signal intensity [30], we trimmed the last 10 bases of reads.

Mapping reads to reference genome was performed using Burrows-Wheeler Alignment tool (BWA ver 0.6.2) [31], with mainly default parameters. SAMtools (ver.0.1.19) [32] was used to convert the alignment results (in SAM format) to BAM format. Duplicated reads were removed using Picard package [32] and then the two BAM files from two libraries for each individual were merged by SAMtools. Reads were realigned around INDELs and base qualities were recalibrated before calling variants using the Genome Analysis Toolkit (GATK, ver 2.4.9) [33], which can greatly improve the sensitivity and specificity in variant calling [34].

#### Variant calling

The chicken SNP information was downloaded from SNP database in NCBI (ftp://ftp.ncbi.nih.gov/snp/organisms/chicken_9031/VCF/, updated in June 11, 2013) [35]. To exclude most false positive variants, we applied a conservative strategy for both INDEL and SNP calling. First, we require a minimum quality score of 20 for both mapped reads and bases to call variants [36]. Then, the SAMtools mpileup and GATK UnifiedGenotyper module were used to call variants independently. The samples were analyzed together. The variants called by both algorithms were retained for further analysis.

#### Post filtering

Stringent filtering criteria were applied to the concordant part of variants using GATK VariantFiltration module. For INDELs, only those meeting all the following criteria were retained: a) read depth between 5 and 31; b) quality by depth (QD)>5.0; c) ReadPosRankSum >−20.0 and d) FS<200.0. For SNPs, the criteria were: a) read depth between 5 and 31; b) QD>5.0; c) MQ>40.0; d) HaplotypeScore <13.0; e) MQRankSum >−12.5; f) ReadPosRankSum >−8.0 and g) FS<60.0. Besides, if more than 3 SNPs were clustered in a 10 bp window, they were all considered as false positives and removed [36]. We also eliminated all the heterozygous variants on sex chromosomes, because in the genome of female chickens, these heterozygous variants can either be caused by error or are within the pseudo-autosomal regions.

### PCR assay validation

To evaluate the reliability of our data, we randomly selected a subset of putative variants for PCR validation in the positive chickens. PCR primers were designed using Primer Premier5 (http://www.premierbiosoft.com/primerdesign/) [37] to amplify the genomic sequences of 250–600 bp containing the variant. Purified PCR products were analyzed with Sanger sequencing as the gold standard.

### Functional annotation

The Ensembl chicken gene set (Ensembl release 74) was downloaded from the Ensembl website (ftp://ftp.ensembl.org/pub/release-74/fasta/gallus_gallus/cdna/) [38] and gene-based annotation of putative INDELs were conducted using ANNOVAR [39]. The chicken QTL database was downloaded from Animal QTL database website (http://www.animalgenome.org/cgi-bin/QTLdb/GG/index, updated in July 8, 2013) [40]. Originally, there were 3807 QTLs in chicken QTL database, but not all of them are suitable for analysis because the confidence intervals of some QTLs were too large to be used efficiently in post-processing. We discarded QTLs with confidence intervals greater than 10 Mb and merged any two or more QTLs with overlapped confidence intervals greater than 50% into one larger QTL. An in-house PERL script was generated to perform the QTL-based annotation. We performed Gene Ontology (GO) functional annotation and Kyoto Encyclopedia of Genes and Genomes (KEGG) pathway analysis on genes affected by INDELs with the DAVID tool (ver 6.7) [41]. We used the default population background for enrichment calculation. Statistical significance was assessed by using *P* value (*P*<0.05) of a modified Fisher's exact test and Benjamini correction for multiple testing.

### Data availability

All raw sequence data had been deposited in NCBI Sequence Read Achieve (SRA) under the Bioproject number PRJNA232548. The experiment numbers for the 12 chickens are SRX408161-SRX408172. The whole variant information was provided in the supplementary files.

## Results

### Sequencing and mapping summary

On average, about 140 million raw reads were generated for each individual, 90% of which were aligned to the reference genome (Table 1). Here, we defined the “effective depth” as the read depth calculated from the reads with mapping quality greater than 20 (Q20). The effective depth ranged from 6.8 in CS to 10.5× in DX, with an average of 8.6×, which was sufficient for further analysis. The overall genome coverage ranged from 94.42 in SG to 95.42% in WL and WR, with an average of 95.02% (Table 1).

**Table 1:** Summary of sequencing and mapping statistics.

Chicken breeds*	Raw reads	Mapped reads (Ratio,%)	Q20 Reads (Ratio,%)	Effective Depth (X)	Coverage (%)
BY	122,734,374	108,899,430(89)	98,055,844(80)	8.2	94.78
CS	113,814,596	102,118,711(90)	81,636,500(72)	6.8	94.67
DX	160,799,966	146,498,490(91)	125,254,482(78)	10.5	95.26
LX	146,127,228	129,219,851(88)	100,947,015(69)	8.4	95.03
RIR	168,078,474	151,117,490(90)	98,330,708(59)	8.2	95.12
RJF	161,325,436	144,056,310(89)	100,985,637(63)	8.4	94.92
SG	141,222,608	124,890,623(88)	82,966,840(59)	6.9	94.42
SK	115,578,334	104,212,249(90)	91,427,763(79)	7.6	94.83
TB	132,544,720	121,217,370(91)	103,736,982(78)	8.7	95.07
WC	143,636,242	132,110,332(92)	114,868,135(80)	9.6	95.24
WL	131,298,592	120,759,421(92)	112,326,911(86)	9.4	95.42
WR	143,375,106	132,693,886(93)	123,918,088(86)	10.4	95.42
Average	140,044,640	126,482,847(90)	102,871,242(73)	8.6	95.02

*Chicken abbreviations: BY, Beijing You; CS, Cornish; DX, Dongxiang; LX, Luxi Game; RIR, Rhode Island Red; RJF, Red Jungle Fowl; SG, Shouguang; SK, Silkie; TB, Tibetan; WC, Wenchang; WL, White Leghorn; WR, White Plymouth Rock.

### INDEL discovery

Although both INDELs and SNPs were identified in our study, we focused on INDELs for further analysis and discussion. In total, 1,766,724 and 1,759,849 raw INDELs were called by SAMtools and GATK, respectively. The concordant part contained 1,425,081 INDELs, accounting for 80.66% and 80.98% of the total number called by SAMtools and GATK, respectively (Figure S1A). In terms of SNPs, 16,153,912 and 16,750,183 raw SNPs were called by SAMtools and GATK, respectively, and the 15,470,364 concordant SNPs corresponded to 95.77% and 92.36% of the two datasets, respectively (Figure S1B). Finally, a huge non-redundant set of variants were obtained after stringent filtering, including 1,343,782 INDELs and 13,708,560 SNPs (Table 2; Table S1; File S1; File S2). The number of INDELs detected in each chicken varied from 368,813 in CS to 528,174 in WR, with an average of 442,794. More than 70% of these variants were detected in two or more individuals.

**Table 2:** Short INDELs detected in 12 diverse chicken breeds.

Chicken breeds^a^	INDEL count			Affected bases (bp)			Novel (Ratio,%)	Maximum length (bp)		Indel Rate (kb^−1^)
	Total	Insertion^b^	Deletion^b^	Total^b^	Insertion^b^	Deletion^b^		Insertion	Deletion	
BY	415,540	196,981	201,852	1,176,135	526,774	649,361	370,997(89.28)	29	47	0.48
CS	368,813	175,456	179,557	1,050,011	470,460	579,551	327,938(88.92)	29	47	0.52
DX	497,358	233,956	241,907	1,439,295	643,553	795,742	445,606(89.59)	29	49	0.45
LX	435,935	205,138	213,611	1,266,168	562,840	703,328	390,120(89.49)	28	45	0.50
RIR	421,309	200,618	203,666	1,219,384	550,048	669,336	375,640(89.16)	29	47	0.49
RJF	451,695	213,427	219,902	1,294,215	582,830	711,385	407,594(90.24)	30	47	0.51
SG	383,782	182,043	186,521	1,092,190	489,868	602,322	342,332(89.20)	29	44	0.53
SK	400,982	189,927	195,966	1,146,582	512,067	634,515	356,250(88.84)	29	45	0.50
TB	448,575	211,393	218,550	1,284,935	572,294	712,641	401,789(89.57)	30	45	0.49
WC	476,889	223,743	233,162	1,384,543	614,443	770,100	427,478(89.64)	29	45	0.47
WL	484,471	229,597	231,861	1,371,739	620,051	751,688	431,757(89.12)	29	44	0.49
WR	528,174	248,212	254,789	1,519,228	681,962	837,266	472,901(89.54)	29	45	0.49
Union^c^	1,343,782	549,806	701,623	3,794,977	1,439,988	2,354,989	1,242,748(92.48)	30	49	1.49

a Chicken abbreviations: BY, Beijing You; CS, Cornish; DX, Dongxiang; LX, Luxi Game; RIR, Rhode Island Red; RJF, Red Jungle Fowl; SG, Shouguang; SK, Silkie; TB, Tibetan; WC, Wenchang; WL, White Leghorn; WR, White Plymouth Rock.

b INDELs that have multiple genotypes were excluded.

c Corrected for INDELs called in more than one individual.

### Assessment of the variant discovery strategy

We compared our results with the variants in SNP database (NCBI dbSNP, updated in June 11, 2013) and found that the vast majority (92.48%) of our INELs were novel (Table 2). The 101,034 concordant INDELs account for 23.02% of the INDELs in the current SNP database. Similarly, about half of the SNPs (48.01%) in our dataset had not been discovered previously (Table S1) and the 7,127,652 concordant SNPs account for 81.07% of all known SNPs in the SNP database.

In order to evaluate the accuracy of our variant detection strategy, we randomly selected 57 INDELs for validation and 50 of them were successfully amplified and sequenced. These INDELs include 23 insertions and 27 deletions, with their sizes ranging from 1 to 31 bp (Table S2). To address the ambiguity of coordinates of some INDELs in repeat regions, we left aligned these INDELs. For example, if the result of whole genome sequencing is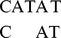, and the Sanger sequencing result may be like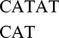. In such case, we assumed that they were different representations of the same allele, and performed left-alignment of INDELs and considered this INDEL as true variation. Finally, 44 of the 50 sequenced INDELs were consistent with the whole genome sequencing results, corresponding to a validation rate of 88.00%. In addition, we also successfully sequenced 44 SNPs from the selected 53 SNPs and obtained an accuracy of 90.91% (40/44) (Table S3).

### Genomic distribution of INDELs

**Figure 1:**
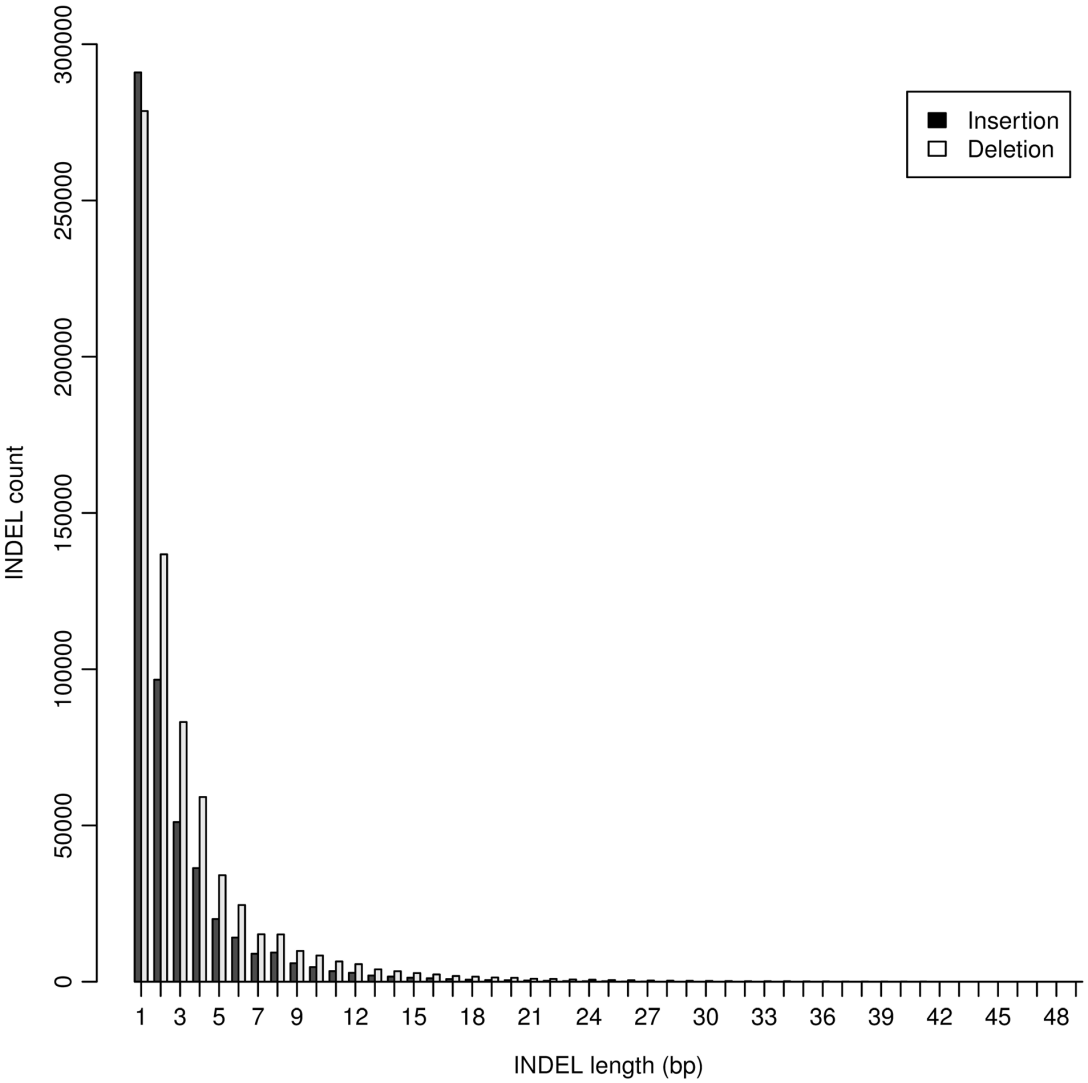
Distribution of INDEL length. INDELs with multiple genotypes were not included.

The largest INDEL detected in this study was 49 bp (Table 2), and the majority (95.76%) of INDELs were less than 10 bp (Figure 1). Single base-pair INDEL was the dominant form and accounted for 45.33% of all detected INDELs. Both the detected number and affected bases were larger for deletions than insertions (Table 2). In total, INDELs affected 3.8 million bases, accounting for 0.36% of the chicken genome.

**Figure 2:**
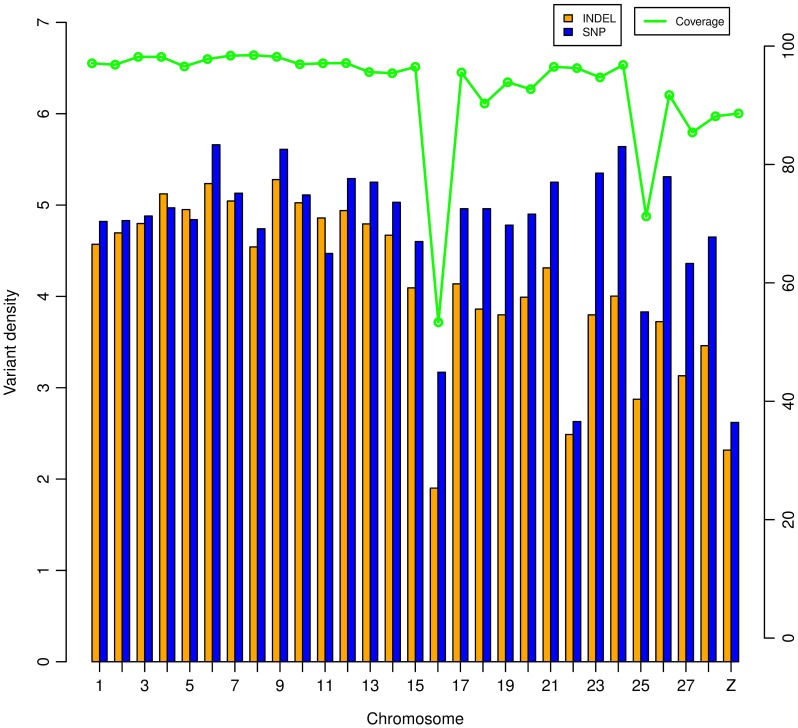
INDEL and SNP density in each chromosome. Densities were calculated as the number per 10 kb (INDEL) and kb (SNP), respectively. Densities are averaged by chicken individuals and corrected by read depth. Coverage was calculated based on Q20 reads.

**Figure 3:**
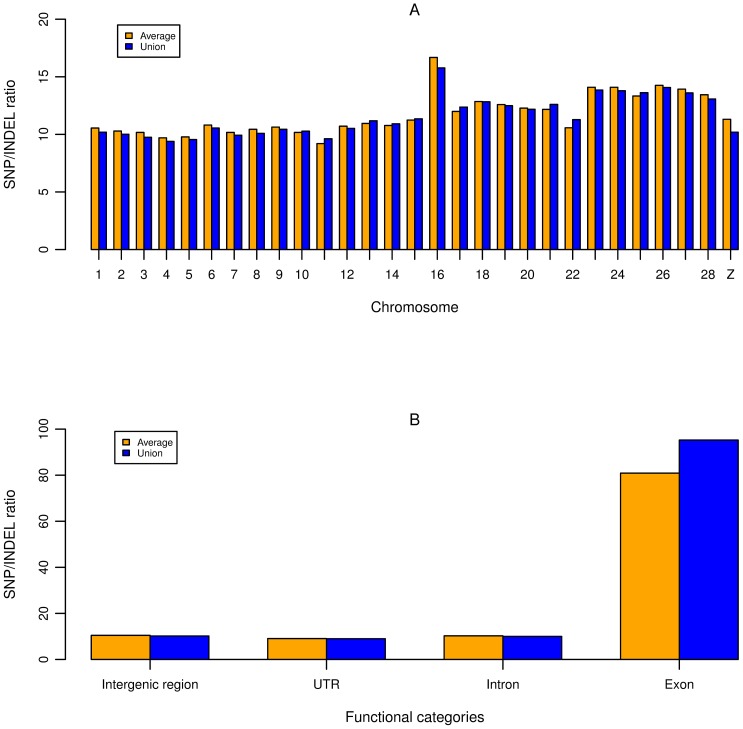
SNP to INDEL ratio. The ratios were plotted based on the non-redundant (Union) data and the data averaged by chickens (Average), respectively. A: SNP to INDEL ratio across chromosomes. B: SNP to INDEL ratios in functional categories.

After correcting the read depth in each individual, we observed the average genomic INDEL density was 0.49 per kb, ranging from 0.45 in DX to 0.53 INDELs per kb in SG (Table 2). We calculated the INDEL density for each chromosome and corrected the density by corresponding read depth. INDELs were distributed in a non-uniform fashion across chromosomes (*P*<2e-16), with INDEL densities of macro-chromosomes (GGA1-5) and intermediate chromosomes (GGA6-10) significantly higher than that of micro-chromosomes (GGA11-28) (0.48, 0.50 vs. 0.38, *P* = 0.0018) (Figure 2). The Z chromosome tended to have lower INDEL density than most autosomes, with its density 45% lower than the average of autosomes. The chromosome 16 was found to have the lowest INDEL density. The SNP to INDEL ratio was calculated and plotted across each chromosome, based on the union and average data, respectively (Figure 3A). Micro-chromosomes tended to have a higher SNP to INDEL ratio, and notably, GGA16 showed the highest ratio, both on average and union (16.67 and 15.74, respectively).

To explore the distribution of INDELs in genic regions, we annotated all detected INDELs using Ensembl gene set (containing 17,954 genes). For each INDEL, its genomic location (intergenic, exonic, intronic, splicing, 5′UTR, 3′UTR, upstream or downstream) and functional role (frameshifting, non-frameshifting and stop gain/loss) were determined. In total, 620,253 (46.15%) INDELs were mapped to genic regions (13,489 genes) (Table 3). Among them, 17,770 INDELs (2.87%) fell in untranslated regions (UTRs), 219 (0.04%) in non-coding transcripts (ncRNAs), 318 (0.05%) in splicing sites, 600,181 (96.76%) in introns, and 1,765 (0.28%) in coding exons. The INDEL densities of intergenic regions, exon, intron and UTR were 0.40, 0.02, 0.45 and 0.09 per kb, respectively. We then examined the SNP to INDEL ratio in these functional regions, and as expected, exon showed the highest ratio (Figure 3B).

**Table 3:** Statistics of INDELs and SNPs in functional regions.

Category	INDEL	Category	SNP
Intergenic	690,303	Intergenic	7,035,013
Flanking region^a^	33,226	Flanking region^a^	345,361
Upstream	13,925	Upstream	163,068
Downstream	18,374	Downstream	171,990
Up/downstream^b^	927	Up/downstream^b^	10,303
Genic	620,253	Genic	6,328,186
5′URT	1,388	5′URT	19,814
3′URT	16,372	3′URT	139,967
5′/3′UTR^c^	10	5′/3′UTR^c^	151
Splicing	318	Splicing	543
ncRNA^d^	219	ncRNA^d^	1,740
Intronic	600,181	Intronic	5,997,846
Exonic	1,765	Exonic	168,125
Non-frameshift	720	Synonymous	119,816
Frameshift	1,022	Non-synonymous	47,915
Stop gain/loss^e^	23	Stop gain/loss^e^	394

a Regions that are 1 kb apart from the transcription start site.

b Variant located in both upstream and downstream regions (possibly for two different genes).

c Variants located in both 5′UTR and 3′UTR regions (possibly for two different genes).

d Variants located in the transcripts without coding annotation in the current Ensembl gene annotation.

e Variants caused gain or loss of stop codon.

In terms of the potential roles of the coding INDELs, 720 (40.79%) were triplet (non-frameshifting), thus retaining the reading frame, and 23 (1.30%) caused gain or loss of stop codon. The remaining 1,022 (57.90%) were non-triplet INDELs, which were predicted to cause frameshift mutation, and this proportion was significantly lower compared with the genomic level (83.55%, *P* = 2.2e-16). A large number (1,358, 7.56%) of functionally important genes were covered by coding INDELs, many (284, 20.91%) of which contained two or more coding INDELs (Table S4).

We then examined the distribution of INDELs in quantitative trait loci (QTL) regions (Table S5). According to our filtering criteria, 595 non-overlapping QTL regions were obtained for analysis. A total of 76,387 INDELs fell into these regions, 37,330 (48.87%) of which located in genic regions. INDEL densities varied significantly across QTL regions, ranging from 0.09 to 3.89 per kb. The average INDEL density for all QTL regions was 1.50 per kb, slightly higher than the genomic level. Several QTLs on GGA4, GGA1, GGA6 and GGA12 that govern feather pecking, chicken body composition, body weight, growth, and abdominal fat percentage had the highest INDEL density.

### Gene enrichment

GO and KEGG pathway analysis were performed on 1,593 genes that contained more than one hundred INDELs, which we assumed to be under high mutation load of INDELs. GO results showed 211 terms, 76 of which were significant after Benjamini correction. These genes were significantly enriched in the molecular functions of protein kinase activity, enzyme activator activity, molecule binding (including nucleotide binding, ion binding), GTPase regulator activity, channel activity, and substrate specific channel activity (Table S6). The KEGG pathway analysis revealed that the genes were overrepresented in 13 pathways, but only one (gga04070: Phosphatidylinositol signaling system) was significant after Benjamini correction.

## Discussion

In this study, we performed NGS on 12 chicken individuals for INDEL discovery to gain a comprehensive understanding of INDEL variation in chicken genome. Although NGS technologies are routinely used to detect genome-wide variations [42], [43], accurately discriminating true variants from false positives from NGS data is still challenging with no easy fix, especially for short INDELs [36], [44], [45]. In this study, we adopted a conservative method to minimize the false positive rate. Several steps that had been proven effective in reducing false positives in variant detection [34], [43], [46]–[48] were adopted in the current study (See Materials and Methods). These measures ensured a significant improvement in detection accuracy compared with a recent study [25] (88.0% vs. 68.4%), even that we had a much lower depth (8.6 vs. 24.9, on average). We anticipate that the combination of advanced sequencing platforms, higher sequencing depth, and superior calling algorithms can further improve the accuracy of INDEL detection in the future. Meanwhile, our method should also suffer a significant false negative rate since we gave much priority to specificity with the sacrifice of sensitivity. For instance, only INDELs called by both algorithms were retained and then subjected to stringent filtering.

To our knowledge, the number of INDELs identified in our study is the highest so far in chicken. Compared with the study in human, this number is smaller than the results from Mills et al. [3], but comparable with a recent study [49], and if we note that the chicken genome is only about one third to human [50], this number will be of great significance. The INDELs accounted for 8.92% of all detected variants and 21.68% in terms of bases involved. These proportions were lower than those observed in other species as described above, and also lower than a recent study in chicken [25], which is probably due to the more stringent filtering criteria and the narrower range of INDEL length in our study. Anyway, our INDELs affected 0.36% of the chicken genome, suggesting that INDELs are widespread in chicken genome and may be an important source of both genetic and phenotypic variation. Over 70% of the 1.2 million INDELs were shared by two or more individuals in spite of their distant genetic relationship, probably representing common variations. Certain unique INDELs may represent the special individual characteristics. The vast majority of detected INDELs were novel, indicating that the discovery of INDELs in chicken, or at least short INDELs, is far from complete. Our results also demonstrated that employing chickens with diverse genetic background for variant detection promoted identifying more variants, as can be seen from the low concordant rate with the INDELs in the SNP database. Hence, for a more comprehensive genetic variation map in the future, multiple individuals and more diverse breeds will be desired.

The INDEL density analyzed in 12 chickens was higher than that observed by Brandstrom and Ellegren [5] because we didn't exclude INDELs in tandem repeat sequence, and also higher than that in human [3]. The Z chromosome had lower INDEL density than autosomes, which was also observed by Brandstrom and Ellegren [5]. This difference would be in part due to the lower effective population size of Z chromosome caused by skewed reproductive success among male chickens [51]. In addition, the lower coverage of Z chromosome than autosomes (88.61% vs 93.03%) and the filtering of heterozygous variants on Z chromosome may also contribute to the lower INDEL density. We observed that the micro-chromosomes tend to have lower INDEL densities, which was consistent with previous results [5]. This may be explained partly by their lower coverage of Q20 reads and partly by the fact that micro-chromosomes are extremely gene rich [50], therefore length mutations, like INDELs, are strongly selected against. In our study, the GGA16 was found to have lower INDEL density than other chromosomes, contrary to previous findings [21], [25]. It could be speculated that this may be caused by the poor coverage of Q20 reads, as well as the partial representation of GGA16 in the current chicken genome assembly. The GGA16 has only been sequenced 535.27 kb, whereas its full length is predicted to be between 9 and 11 Mb [52]. In spite of the low density of INDELs and SNPs on GGA16, the SNP to INDEL ratio in GGA16 was the highest among all chromosomes. This may result from the presence of several important gene families, like nucleolus organizer region (NOR) and major histocompatibility complex (MHC), an immune-related gene family, which could impose a greater selection pressure on INDELs than SNPs. As mentioned above, INDELs as a kind of length variants are often deleterious to gene functions, whereas SNPs generally cause little or no effects to gene functions. Besides, the SNP to INDEL ratio was strikingly high in exons, and both the INDEL density and the proportion of frameshifting INDELs was significantly lower than that of genomic level. This indicated that INDELs in exons, frameshifting INDELs in particular, were strongly eliminated by purifying selection. We also found that INDELs were enriched in some QTLs, which was likely due to the recent selection for favorable alleles. These INDELs could be used as candidate markers for fine mapping of causative genes.

Like SNPs and CNVs, INDELs are of great importance for their ability to alter gene functions, especially those frameshifting INDELs locating in exons. In this study, lots of genes were affected by frameshifting INDELs. Some genes are associated with chicken performance traits. For instance, *THRSP* encodes a small acidic protein that responds to thyroid hormone (TH) stimulation and is thought to play a role in growth. A 9 bp INDEL polymorphism and several SNPs in the exon1 of *THRSP* were found to be associated with abdominal fat content [53], [54] and body weight [55]. In our study, two novel INDELs within the exon1 were found in several chickens, implicating that this gene was highly polymorphic and the two novel INDELs were worth further studying for their association with economic traits. *MUC6* (β-subset of ovomucin) is the homologue of human *MUC6* [56] and reported to be involved in determining the gel property of thick egg white [57]. As many as five coding INDELs were identified in *MUC6* and we suggested that these INDELs could be used as potential candidates for egg quality. In addition, quite many genes related to the development of chicken embryo or are the homologues of human disease-related genes. The results demonstrated that though strongly selected against, INDELs were common in some functionally important genes, arguing for their incorporation to elucidate the association between genes and traits.

It is increasingly recognized that INDEL polymorphisms can be effectively used as genetic markers [16], [58]–[60]. In fact, INDELs merit as promising genetic markers for many aspects. First, INDELs are diallelic and widespread throughout chicken genome. The density of INDELs in chicken genome is much higher than that of microsatellite [61], which can compensate their shortcoming of lower level of polymorphism. Second, INDELs are relatively easy and cost-effective to genotype, allowing ordinary laboratories to rapidly screen a large number of individuals [16]. Third, the probability of two INDELs of the same length occurring at the same position is very low that the shared INDELs can confidently be related to identity by descent [62]. This can reduce the occurrence of the homoplasy, a common problem in phylogenetic studies using microsatellites as markers. Forth, most INDELs have a minor allele frequency (MAF) greater than 0.05 [3], [17], [58], [60], meeting the criteria of common genetic variations. Finally, most INDELs are in strong linkage disequilibrium (LD) with SNPs of genome-wide association studies (GWASs) [3], suggesting that INDELs are likely to associate with a substantial amount of phenotypic diversity and disease susceptibility. Therefore, INDELs can be efficiently integrated into current genetic variation map to construct a more comprehensive map including SNPs, INDELs and CNVs, which will facilitate the identification of causative mutations and accelerate genetic improvement for complex traits and diseases.

Microarrays are very powerful and essential tools in GWAS and genomic selection (GS). Up to date, medium and high density SNP arrays have been commercially available [63], [64] in chicken, whereas no INDEL array is reported available not only in chicken but also in any other domestic animals. Efforts to design INDEL arrays have been made by Salathia et al. [65] and Mills et al. [3] in Arabidopsis thaliana and human, respectively. Though both arrays contained a relatively small number of INDELs, they shed light on the feasibility of designing INDEL arrays and genotyping large number of individuals. Currently, the paucity of available INDEL resources may hamper the development process since developing INDEL arrays requires a large collection of polymorphic INDELs. The large quantity of INDELs screened in our study enriched the current INDEL database and will be beneficial to future development of INDEL arrays. In addition, researchers can also select a number of informative INDELs and integrate them into SNP arrays to increase their power in GWAS and GS.

## Conclusions

We performed whole genome sequencing on 12 diverse chicken breeds and identified the largest number of INDELs in chicken genome so far. Incorporating diverse chicken breeds for variant detection allowed for a larger collection of variants to be discovered. A large number of coding INDELs located in previously reported genes associated with chicken performance traits. We suggest that INDELs are crucial determinants causing genetic and phenotypic diversity and can be promising genetic markers. Our results can be used for a variety of studies in the future, including development of INDEL markers, construction of high density linkage map, INDEL arrays design, and hopefully, molecular breeding programs in chicken.

## Supporting Information

Figure S1 **The number of raw variants called by SAMtools and GATK, respectively.** A: INDELs. B: SNPs.
(TIFF)

Table S1 **SNPs detected in 12 chickens.**
(DOCX)

Table S2 **Summary of validated INDELs.**
(XLSX)

Table S3 **Summary of validated SNPs.**
(XLSX)

Table S4 **Coding INDELs in each chromosome.**
(XLSX)

Table S5 **Distribution of INDELs in QTL regions.**
(XLSX)

Table S6 **Functional enrichment of genes under high mutation load of INDELs.**
(XLSX)

File S1 **The non-redundant set of detected INDELs in 12 chickens.** To minimize the file size, only minimal essential information was listed, including CHR (chromosome), POS (position), REF (reference allele), ALT (alternative allele), and BREEDS (chickens in which this INDEL was detected).
(GZ) Click here for additional data file.

File S2 **The non-redundant set of detected SNPs in 12 chickens.** To minimize the file size, only minimal essential information was listed, including CHR (chromosome), POS (position), REF (reference allele), ALT (alternative allele), and BREEDS (chickens in which this SNP was detected).
(GZ) Click here for additional data file.
