# Supplementary figures and images for: Genome-Wide Characterization of Insertion and Deletion Variation in Chicken Using Next Generation Sequencing
Source: PLoS One. 2014 Aug 18;9(8):e104652. doi: 10.1371/journal.pone.0104652 (PMC4136736; doi:10.1371/journal.pone.0104652)

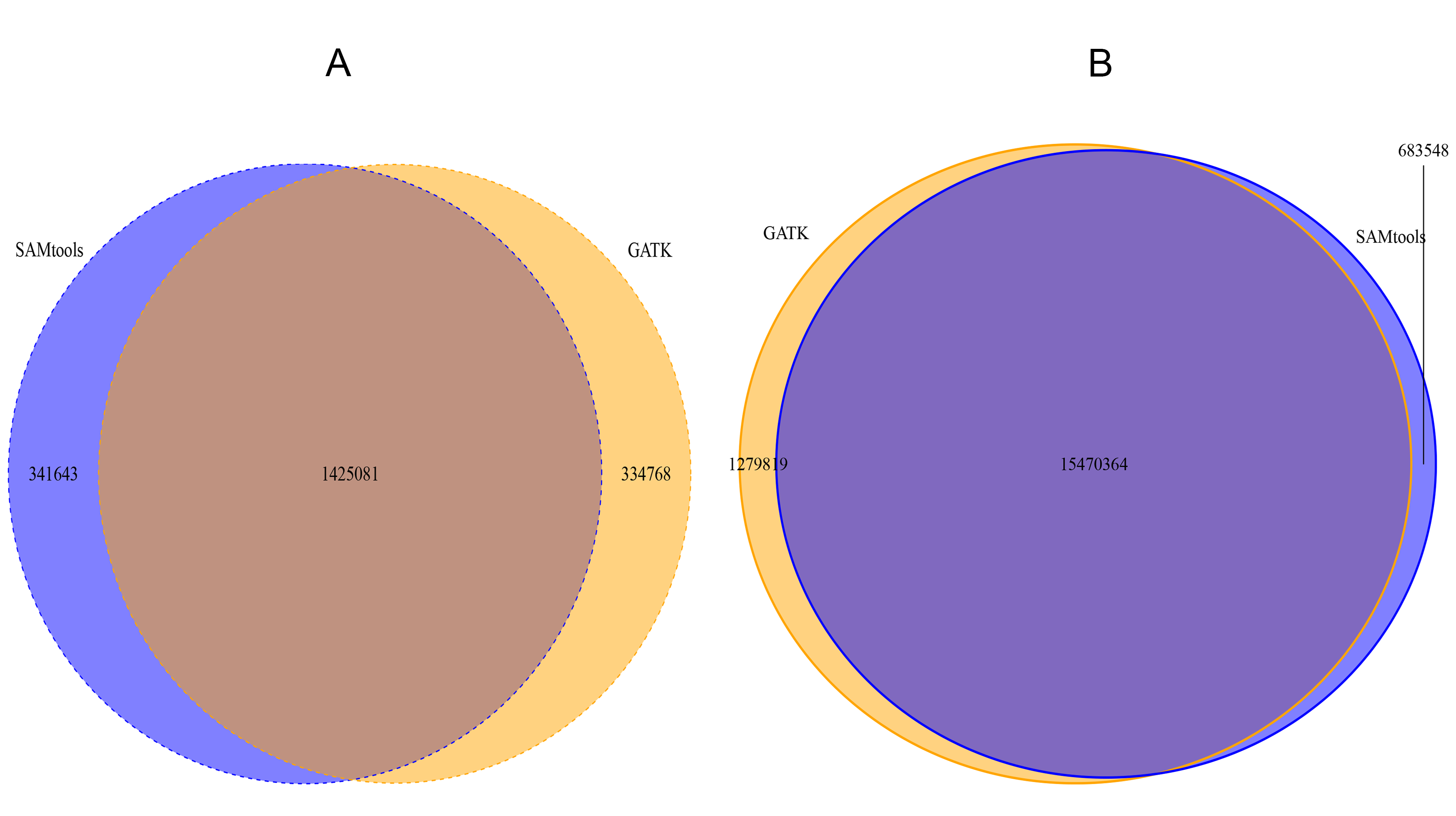

Supplement: Figure S1 — The number of raw variants called by SAMtools and GATK, respectively. A: INDELs. B: SNPs. (TIFF) [file pone.0104652.s001.tiff]
