# Supplementary material for: Genome-Wide Characterization of Insertion and Deletion Variation in Chicken Using Next Generation Sequencing
Source: PLoS One. 2014 Aug 18;9(8):e104652. doi: 10.1371/journal.pone.0104652 (PMC4136736; doi:10.1371/journal.pone.0104652)
Supplement: Table S1 — SNPs detected in 12 chickens. (DOCX) [file pone.0104652.s002.docx]

Table S1 SNPs detected in 12 chickens

| Breeds^a^ | Total | Novel SNP (Ratio, %) | Multiallelic SNP | Het/Hom^b^ | Ti/Tv^c^ | Density (kb^-1^) |
| --- | --- | --- | --- | --- | --- | --- |
| BY | 3,915,477 | 1,055,330(26.95) | 5,065 | 0.63 | 2.53 | 4.56 |
| CS | 3,546,773 | 852,675(24.04) | 4,459 | 0.63 | 2.56 | 4.98 |
| DX | 5,231,614 | 1,623,981(31.04) | 6,889 | 0.89 | 2.45 | 4.76 |
| LX | 4,260,282 | 1,246,172(29.25) | 6,772 | 0.97 | 2.55 | 4.84 |
| RIR | 3,926,735 | 981,866(25.00) | 5,271 | 0.65 | 2.55 | 4.57 |
| RJF | 5,196,536 | 1,966,868(37.85) | 8,192 | 1.21 | 2.43 | 5.91 |
| SG | 4,201,943 | 1,237,492(29.45) | 5,073 | 0.79 | 2.41 | 5.82 |
| SK | 4,653,522 | 1,402,983(30.15) | 6,396 | 0.96 | 2.46 | 5.85 |
| TB | 5,030,722 | 1,569,953(31.21) | 7,422 | 1.22 | 2.44 | 5.52 |
| WC | 5,390,279 | 1,699,396(31.53) | 8,290 | 1.39 | 2.45 | 5.36 |
| WL | 4,971,630 | 1,274,027(25.63) | 5,388 | 0.76 | 2.47 | 5.05 |
| WR | 5,234,898 | 1,430,915(27.33) | 6,145 | 0.82 | 2.46 | 4.81 |
| Union^d^ | 13,708,560 | 6,580,908(48.01) | 77,300 | - | 2.48 | 15.23 |

^a^ Chicken abbreviations: BY, Beijing You; CS, Cornish; DX, Dongxiang; LX, Luxi Game; RIR, Rhode Island Red; RJF, Red Jungle Fowl; SG, Shouguang; SK, Silkie; TB, Tibetan; WC, Wenchang; WL, White Leghorn; WR, White Plymouth Rock.

^b^ Heterozygous to homozygous ratio.

^c^ Transition to tranversion ratio.

^d^ Corrected for SNPs detected in more than one individual.
